# Rapamycin-mediated mTOR inhibition impairs silencing of sex chromosomes and the pachytene piRNA pathway in the mouse testis

**DOI:** 10.18632/aging.101740

**Published:** 2019-01-13

**Authors:** Zhiping Zhu, Qiuling Yue, Jie Xie, Shuya Zhang, Wenxiu He, Shun Bai, Suwen Tian, Yingwen Zhang, Mengneng Xiong, Zheng Sun, Chaoyang Huang, Yuebei Li, Ke Zheng, Lan Ye

**Affiliations:** 1 State Key Laboratory of Reproductive Medicine, Nanjing Medical University, Nanjing, China; 2 The People's Hospital of Gaochun, Nanjing, China; 3 Department of Medicine, Baylor College of Medicine, Houston, TX 77030, USA; 4 Heart and Vascular Center, The First Affiliated Hospital, School of Medicine, Zhejiang University, Hangzhou 310003, Zhejiang, China; 5 The First Medical School of Nanjing Medical University, Nanjing, China; * Equal contribution

**Keywords:** rapamycin, mTOR inhibition, germ cells, meiosis, meiotic silencing of sex chromosomes

## Abstract

Mechanistic target of rapamycin (mTOR) controls cell growth and metabolism in response to environmental and metabolic signals. Rapamycin robustly extends the lifespan in mammals and has clinical relevance in organ transplantation and cancer therapy but side effects include male infertility. Here, we report that chronic rapamycin treatment causes spermatogenic arrest in adult male mice due to defects in sex body formation and meiotic sex chromosome inactivation (MSCI). Many sex chromosome-linked genes were up-regulated in isolated pachytene spermatocytes from rapamycin-treated mice. RNA-Seq analysis also identified mRNAs encoding the core piRNA pathway components were decreased. Furthermore, rapamycin treatment was associated with a drastic reduction in pachytene piRNA populations. The inhibitory effects of rapamycin on spermatogenesis were partially reversible, with restoration of testis mass and sperm motility within 2 months of treatment cessation. Collectively, we have defined an essential role of mTOR in MSCI and identified a novel function as a regulator of small RNA homeostasis in male germ cells.

## Introduction

Mechanistic target of rapamycin (mTOR) is a conserved serine/threonine protein kinase that regulates cell survival and metabolism in response to extra- and intracellular signals that include nutrients, growth factors, cellular energy, and stress. mTOR resides in two structurally and functionally distinct multiprotein complexes, mTORC1 and mTORC2 [1–4]. The mTORC1 complex requires the mTOR-associated adaptor protein Raptor and activates ribosomal biogenesis and protein translation by phosphorylation of the ribosomal protein S6 kinase 1 (S6K1) and eukaryotic initiation factor 4E-binding protein 1 (4E-BP1) [5,6]. The mTORC2 complex incorporates the adaptor protein Rictor and exerts metabolic control through AKT and serum/glucocorticoid induced kinase (SGK) [7,8]. mTORC2 also regulates the cytoskeleton through protein kinase C alpha (PKC-α) signaling [9].

The mTOR inhibitor rapamycin exerts diverse biological effects, including immunosuppression and inhibition of proliferation, via binding to immunophilin FKBP12, which results in mTOR inhibition. Consequently, rapamycin and its analogs (rapalogs) have been used to prevent rejection after organ transplantation [10]. Activation of mTOR signaling has been associated with cancer pathogenesis, prompting investigation of rapamycin and rapalogs for the treatment of cancers [11,12]. Currently, rapamycin is FDA approved as immunosuppressant, and its analogs Temsirolimus and Everolimus have been approved for the treatment of advanced-stage renal cell carcinoma and Tuberous Sclerosis complex genetic disease, respectively [1]. Intriguingly, rapamycin is the only small molecule that robustly extends the life span of genetically heterogeneous mice [13]. However, male patients who received rapamycin in post-transplantation and cancer therapies have a high prevalence of infertility, suggesting a critical role of mTOR signaling in spermatogenesis [14]. Furthermore, male mice receiving a rapamycin-supplemented diet in longevity studies exhibited testicular degeneration [15]. Rapamycin influences the self-renewal potential of cultured spermatogonial stem cells (SSCs) and prevents spermatogonial differentiation in neonatal mice [16,17]. In contrast, hyperactivation of mTORC1 signaling by conditional deletion of the mTORC1 inhibitor *Tsc2* in undifferentiated spermatogonia depletes their self-renewal potential, promoting germ cell differentiation and deterioration [18]. However, whether rapamycin affects meiosis and gem cell development in mammals still remains to be defined.

Endogenous small interfering RNAs(endo-siRNAs), microRNAs (miRNAs) and PIWI-interacting RNAs (piRNAs) are three main classes of small non-coding RNAs. mTOR signaling regulates small RNA biogenesis and stability in somatic cells [19]. A recent study found that modification of mTOR signaling by phosphorylation of the upstream negative regulator TSC1 or by targeted mutation of the essential mTORC1 component Raptor influences the biogenesis of precursor and mature microRNAs (miRNAs) through Mdm2-mediated ubiquitination of Drosha [20]. Warner and colleagues identified the underlying molecular link, finding that post-translational modification of the miRNA biogenesis machinery is mediated by TRBP, which is phosphorylated at serines 283/286 by S6K1, a downstream kinase of mTORC1 [21]. Additionally, previous studies have shown that individual miRNAs target mTOR signaling. For example, miR-214 suppresses phosphorylation of PRAS40, S6K, and 4EBP-1, and miR-15b/16 affects availability of the mTORC2 component Rictor by targeting its 3'UTR region [22,23]. These studies suggest that mTOR plays an essential role in maintaining miRNA homeostasis, and furthermore, imply cross-talk between these two pathways. piRNAs are a distinct class of small non-coding RNAs specifically expressed in mouse testes. Interestingly, the piRNA pathway regulates germline-specific alternative splicing of TOR (let-363), a homolog of mammalian mTOR in *C. elegans*, suggesting a potential interaction between the mTOR and piRNA pathways [24]. piRNAs are 24-33 nucleotides(nt) in length, and their biogenesis involves the primary and secondary pathways. Primary piRNA biogenesis includes transcription of primary piRNA precursors from piRNA clusters, cleavage of piRNA precursors into piRNA intermediates, and further trimming into mature piRNAs [25–35]. Three mouse PIWI proteins MILI, MIWI and MIWI2 in the mouse testes are loaded with piRNAs and exert their diverse functions [36–39] including silencing transposons.

Here, we report that chronic rapamycin treatment causes spermatogenic arrest in male mice due to defects in meiotic sex chromosome inactivation. Furthermore, we have identified a novel role of mTOR signaling in maintaining piRNA homeostasis in male germ cells. The inhibitory effects of rapamycin on spermatogenesis are reversible within 2 months following treatment cessation, identifying rapamycin treatment as potential pharmacologic approach for male contraception.

## RESULTS

### Chronic rapamycin exposure disrupts mTOR complex integrity and impairs spermatogenesis

**Figure 1:**
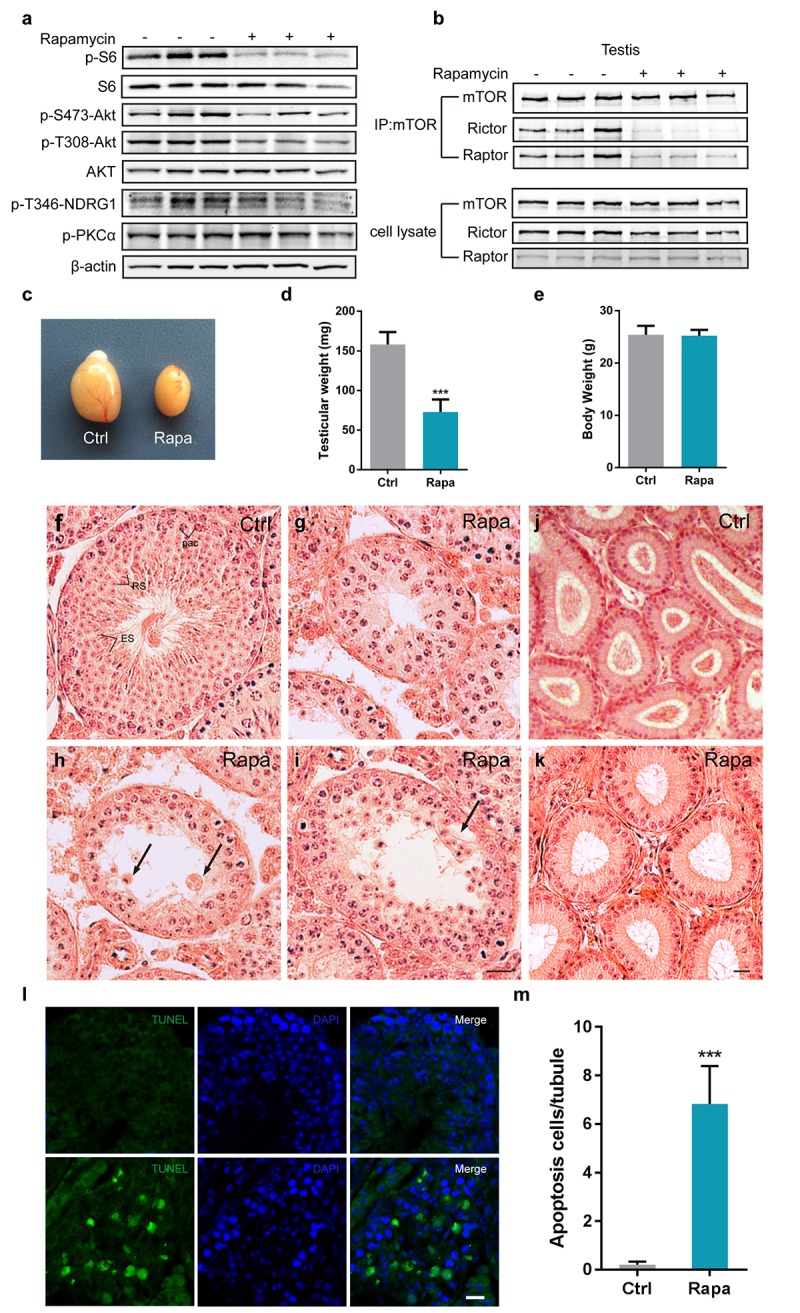
**Chronic rapamycin treatment disrupts spermatogenesis in male mice and inhibits assembly of mTOR complexes.** Tissues from adult males were analyzed after weeks of daily i.p. injection with rapamycin or vehicle control beginning at age 7-8 weeks. (**a**) Western blot analysis of phosphorylated S6, AKT, PKCα, and the SGK substrate NDRG1 in testicular extracts from adult males (control, n=3; rapamycin, n=3). (**b**) Chronic rapamycin treatment impairs mTOR complex integrity and activity. Immunoblotting of mTOR immunoprecipitates from testis tissue from adult males (control, n=3; rapamycin, n=3). (**c**) Gross morphology of testis tissue from control or rapamycin (rapa) treated males. (**d**) Testis weight (control, n=5; rapamycin, n=4). Error bars represent SD (***P < 0.001, Student’s t test). (**e**) Body weight (control, n=5; rapamycin, n=4). (**f-i**) Testis histology. Hematoxylin/eosin stained representative testis sections. Scale bar,20μm.(**f**) Seminiferous tubule from control testis, containing pachytene spermatocytes (Pac), round spermatids(RS), and elongating spermatids (ES), indicating normal spermatogenesis. (**g**) Seminiferous tubule from rapamycin-treated testis with meiotic arrest at the pachytene stage. (**h**) Seminiferous tubule from rapamycin-treated testis with clusters of aggregated round spermatids. Black arrow indicates multinucleated cells. (**i**) Rapamycin-treated tubules with large vacuoles in the seminiferous epithelium. Black arrowhead points to vacuoles. (**j, k**) Histological analysis of epididymis from adult control and rapamycin-treated mice. (**l**) TUNEL staining of testis sections from control and rapamycin-treated mice. Scale bar, 20μm. (**m**) Quantification of TUNEL-positive cells per tubule. Error bars represent SD (**P < 0.01, Student’s t test).

To explore the role of mTOR in spermatogenesis, we treated adult male C57BL/6 mice daily for 3 weeks with rapamycin at the dose shown to extend lifespan (2mg/kg per day; starting at the age of 7 to 8 weeks) or with vehicle control. As expected from the known inhibitory role of rapamycin on mTORC1, rapamycin inhibited the phosphorylation of S6, a downstream substrate of the mTORC1 substrate S6K1 in testis (Fig.1a). Phosphorylation of mTORC2 substrates, including AKT at Serine (S) 473, protein kinase PKCα S657, and the serum glucocorticoid induced protein kinase(SGK) substrate NDRG1 T346 was also decreased (Fig. 1a), consistent with our previous finding that chronic rapamycin exposure disrupted the activity of mTORC2 in the mouse liver [40]. We next immunoprecipitated mTOR from testes tissue and found that association of mTOR with Raptor was almost absent and association with Rictor greatly reduced (Fig.1b), indicating that chronic rapamycin exposure disrupted the assembly of both mTORC1 and mTORC2 in the testis.

Chronic rapamycin exposure resulted in a drastic reduction of mouse testis size (Fig. 1c). Testes from rapamycin-treated mice weighed less than half (72.88 ± 7.876 mg/pair; ~46%) than control testes (158.1 ± 6.980 mg/pair), whereas overall body size was not affected (Fig.1d, e). In contrast to control seminiferous tubules with a full spectrum of spermatogenic cells at various developmental stages (Fig. 1f), testis tubules from rapamycin-treated mice exhibited defects in spermatogenesis, with a predominant arrest of spermatocytes at the pachytene stage (Fig. 1g), suggesting meiotic arrest during meiotic prophase I. Some spermatocytes survived through meiotic prophase I and developed into round spermatids, following by aggregation into large cell clusters (Fig.1h). A subset of tubules contained large vacuoles (Fig. 1I). Histological examination of epididymides from rapamycin-treated mice revealed the absence of mature spermatozoa (Fig. 1j, k) confirming that chronic rapamycin exposure results in infertility. TUNEL analysis revealed dramatically increased apoptosis in rapamycin-treated tubules (Fig. 1l, m), indicating that abnormal spermatocytes were eliminated by apoptosis.

### X-Y assembly defects in chronic rapamycin-treated testes

**Figure 2:**
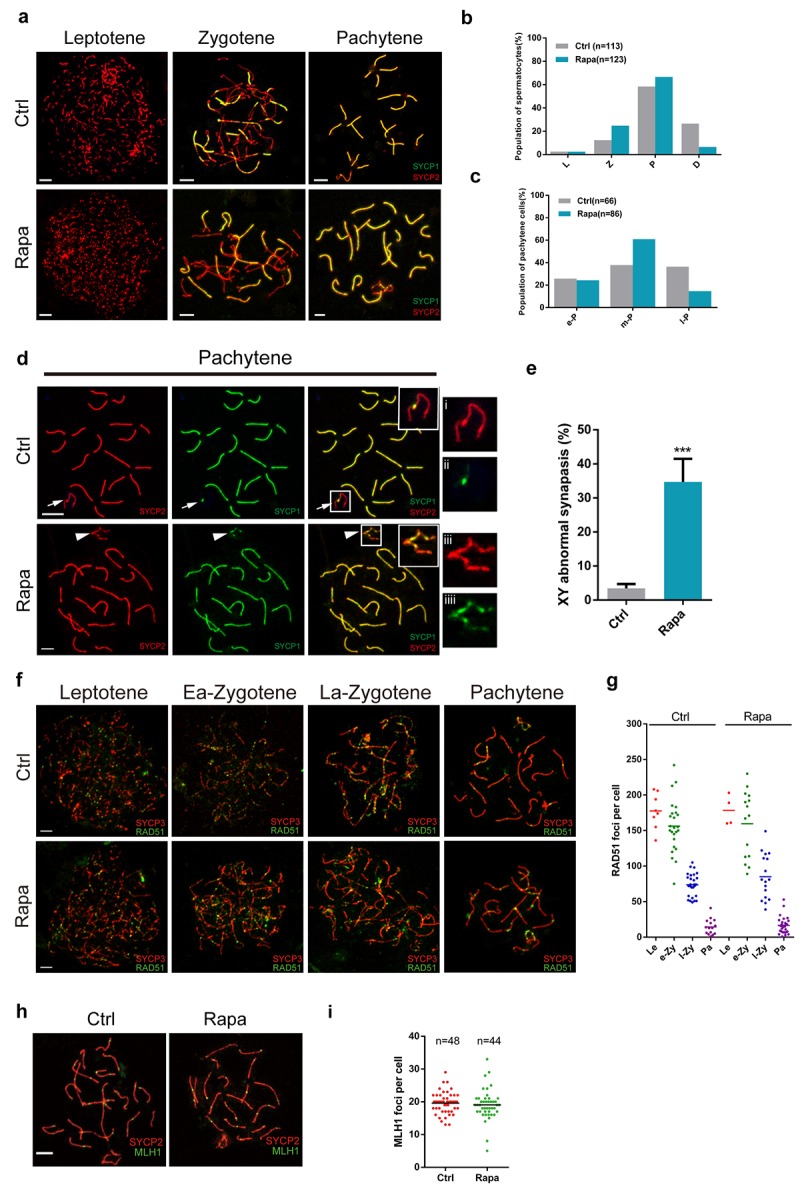
**Meiotic defects in rapamycin-treated spermatocytes.** Immunostaining of spread nuclei of spermatocytes for the synaptonemal complex proteins SYCP3 (red) and SYCP1 (green) or recombination proteins (green). (**a**) Identification of leptotene, zygotene and pachytene spermatocytes by SYCP1 and SYCP3 immunostaining. (**b**) Proportion of meiotic spermatocyte populations in control and rapamycin-treated mice. Leptotene (L), zygotene (Z), pachytene (P) and diplotene (D). (**c**) Percentage of e-P (early pachytene), m-P (middle pachytene) and l-P (late pachytene) spermatocytes. (**d, e**) Significantly increased frequency of abnormal localization of SYCP1 to the sex chromosomes in pachytene stage spermatocytes. White arrows and white arrowheads mark the sex chromosomes in control and rapamycin-treated spermatocytes, respectively. (**f, g**) Immunostaining for RAD51 (green) and SYCP3 (red) in leptotene, early zygotene (Ea-Zygotene), late zygotene (La-Zygotene) and pachytene stage spermatocytes identifies similar numbers of RAD51 foci per cell in spermatocytes from control and rapamycin-treated mice. (**h**) Similar numbers of MLH1 foci per cell in control and rapamycin-treated spermatocytes based on MLH1 (green) and SYCP3 (red) immunostaining. Error bars represent SD (*P < 0.05, Student’s t test).

Rapamycin-treated testis contained arrested spermatocytes at the pachytene stage and exhibited substantial loss of post-meiotic cells, implying meiotic defects. To monitor meiotic progression, we therefore first assessed chromosomal synapsis in rapamycin-exposed spermatocytes by immunofluorescence analysis of spread nuclei using antibodies against SYCP3, a component of synaptonemal complex (SC) lateral elements, and SYCP1, a component of SC transverse elements [41–45]. In control leptotene spermatocytes, SYCP3 immunostaining identified lateral elements, with signal manifesting as multiple short threads, followed by extension of signal at the zygotene stage, when lateral elements become associated via the transverse element SYCP1 (Fig. 2a). With the progression of synapsis, all chromosomes synapsed at the pachytene stage except for the X-Y chromosomes, which normally only pair at a short stretch of SC termed pseudoautosomal region (Fig. 2a). Rapamycin-treated testes contained a larger proportion of zygotene and early pachytene spermatocytes, and a reduced proportion of diplotene and late pachytene spermatocytes than controls (Fig. 2b, c). The distribution of SYCP1 and SYCP3 on autosomes in spermatocytes from rapamycin-treated mice was similar to controls but there was an approximately 9-fold increase in SYCP1 localization to the unsynapsed regions of the X and Y chromosomes (Fig. 2d, e; control, 3.5%, n=210; rapamycin, 34.7%, n=250. χ^2^ test). Similar abnormalities have also been observed in *Ago4* knockout mice [46], which exhibit a dramatic loss of microRNAs.

Chromosome synapsis is tightly associated with mammalian meiotic recombination, which initiates with the formation of meiotic associated double-strand breaks (DSBs), followed by DSB repair and segregation of homologous chromosomes. The recombinase complex RAD51/DMC1 accumulates on DSB sites and facilitates strand invasion for homologue search [47,48]. We evaluated the formation of recombination foci in control and rapamycin-treated spermatocytes, and found a similar number and distribution of RAD51 foci, which were abundant in leptonema and zygonema, followed by reduction in late zygonema, and retention of few foci on autosomal and sex chromosomes at the pachytene stage (Fig. 2f, g). MLH1 is a late marker of the recombination process and marks sites of crossovers at the mid-late pachytene stage. We did not detect a statistically significant difference in the number of MLH1 foci between control and rapamycin-treated pachytene cells (Fig. 2h and i). These results suggest that the formation of recombination intermediates was not impaired by mTOR inhibition.

### Chronic rapamycin exposure alters the localization of essential sex body components

**Figure 3:**
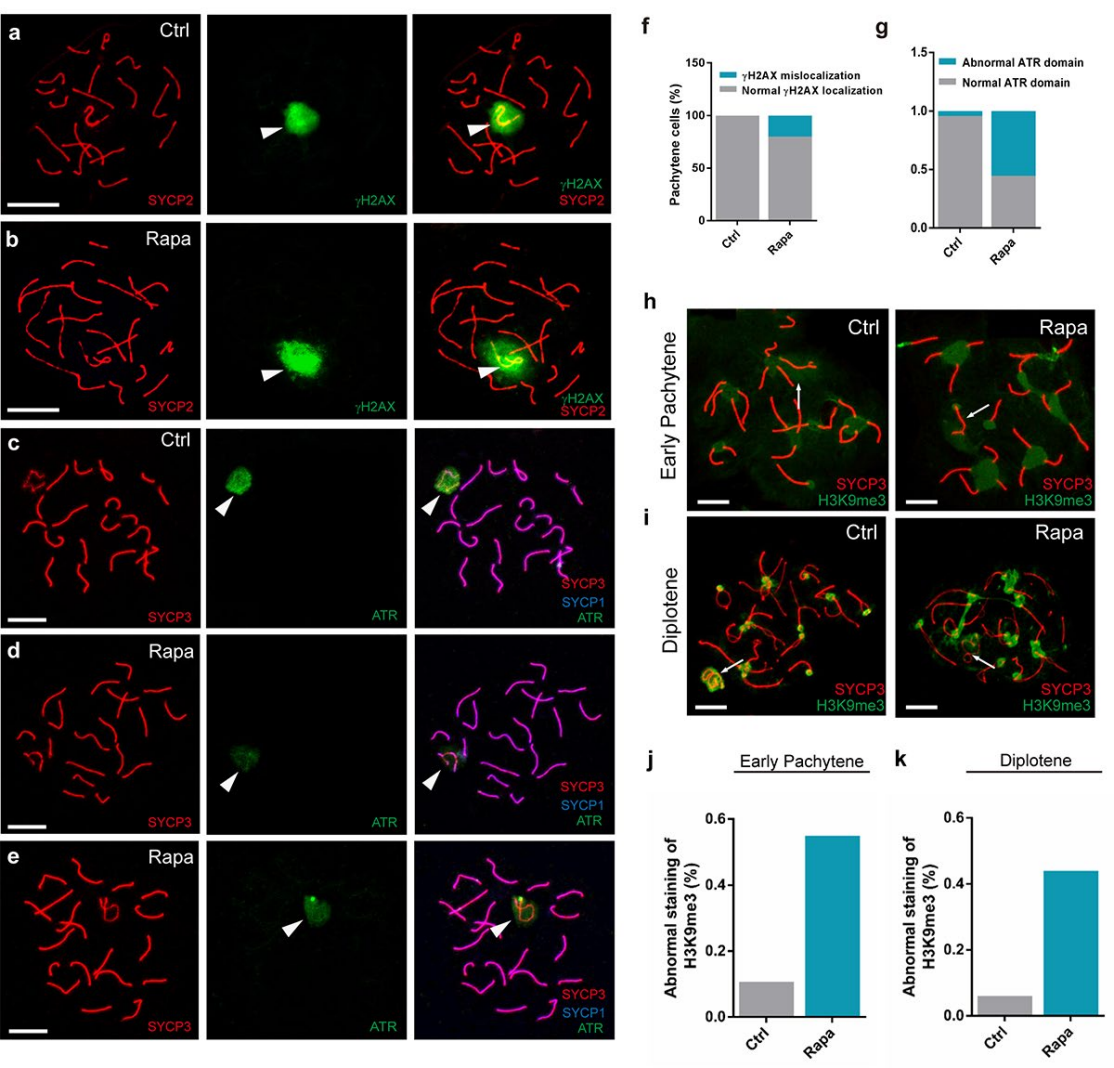
**Abnormal localization of sex body silencing factors in pachytene spermatocytes from rapamycin-treated mice.** Immunostaining of spread nuclei for SYCP3 (red) and silencing factors (green). (**a**) Control pachynema with restriction of γH2AX signal to the sex chromatin. (**b**) Pachynema from rapamycin-treated mice with γH2AX signal in autosomal regions (spermatocytes analyzed: Control, n=210; Rapamycin, n=250. χ^2^ test). White arrowheads indicate the sex chromosomes. (**c-e**) Localization of ATR to sex body and chromosomal axes in control spermatocytes (**c**) contrasting with weak ATR staining to the sex body in spermatocytes from rapamycin-treated mice (d-e). White arrowheads indicate the sex chromosomes. (**f, g**) Percentage of pachytene spermatocytes with abnormal localization of γH2AX or ATR. (**h, i**) Immunostaining for H3K9me3 in early pachytene stage (**h**) and diplotene stage (**i**) spermatocytes from control and rapamycin-treated mice. White arrows mark the sex chromosomes. (**j, k**) Quantification of spermatocytes with defects in H3K9me3 localization at the early pachytene stage (**j**) and diplotene stage (**k**). Scale bar, 10μm.

Sex body integrity is essential for the meiotic silencing of sex chromosomes [49]. Prompted by our observations that rapamycin-exposed spermatocytes exhibited meiotic arrest and abnormal distribution of SYCP1 to the sex chromosomes, we next investigated potential disruptions to the recruitment of sex body components. The formation of DSB triggers recruitment of the serine/threonine kinases Ataxia telangiectasia-mutated (ATM) and its Rad3-related protein (ATR) to unsynapsed chromosomes to phosphorylate H2AX [50–52], causing gene silencing. Previous studies suggest that, during meiotic silencing, the formation of phosphorylated (γ) H2AX is dependent on ATR and is required for gene silencing [53]. In normal spermatocytes, γH2AX accumulates on the unsynapsed regions of chromosomes during the leptotene and zygotene stages of prophase I. Following meiotic DSB repair, γH2AX disappears from the autosomal axes and becomes restricted to the unsynapsed sex chromosomes during the pachytene stage [54] (Fig. 3a). In contrast, in rapamycin-exposed pachynema, γH2AX immunostaining expanded beyond the sex body and was detectable on autosomes in a large proportion of pachytene spermatocytes (Fig. 3b, f; control: 0%, n=200; rapamycin: 20%, n=200). We next examined the localization of ATR, which catalyzes H2AX phosphorylation. In control pachytene spermatocytes, strong ATR signal was present throughout the sex body, and aggregated on the axes of the sex chromosomes (Fig. 3c). Intriguingly, in pachytene spermatocytes from rapamycin-treated mice, ATR was dramatically reduced but with similar distribution (Fig. 3d, e, g; control: 4.3%, n=55, rapamycin: 55.3%, n=60).

To determine whether the abnormal localization of sex body components in rapamycin-treated spermatocytes affected the establishment of various epigenetic modifications that occur during meiotic chromatin remodeling, we next compared the localization of trimethylation of H3K9 (H3K9me3) in testicular cells from control and rapamycin-treated mice. H3K9me3 is a repressive epigenetic marker implicated in meiotic silencing that appears in early pachytene spermatocytes and accumulates on the entire sex chromatin and the centromeres of autosomes (Fig. 3h) [55]. However, the X chromosomes of a large proportion of pachynema from rapamycin-treated mice were devoid of H3K9me3 marks except for the centromeric regions (Fig. 3h, j; control: 6%, n=55, rapamycin: 44%, n=60), whereas distribution of H3K9me3 to the Y chromosome was similar to control. Exclusion of H3K9me3 from the X chromosome was also observed in rapamycin-treated diplotene spermatocytes that escaped from pachytene arrest (Fig. 3i, k; control: 10.7%, n=52, rapamycin: 54.9%, n=61). Taken together, chronic rapamycin exposure impaired the accumulation of sex body components and caused defects in establishment of epigenetic modifications on the sex body.

### Upregulation of sex chromosome-linked genes in rapamycin-treated germ cells

**Figure 4:**
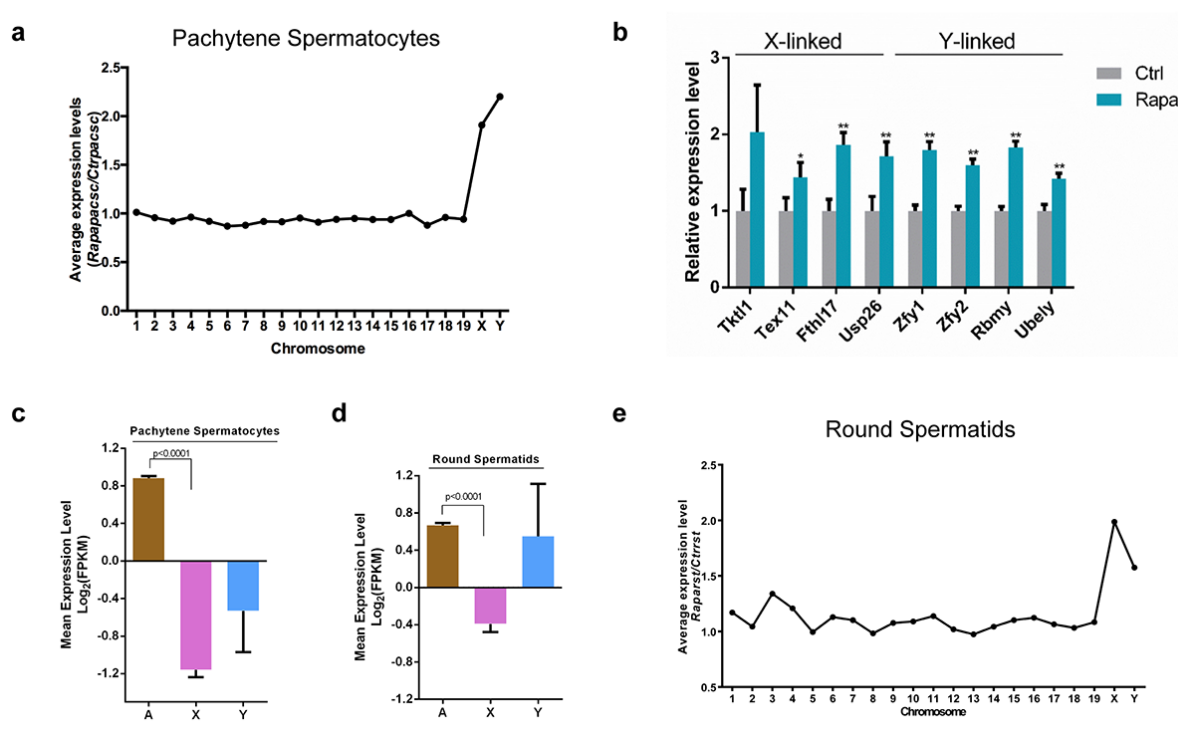
**Transcriptional upregulation of sex chromosome-linked genes in pachytene spermatocytes from rapamycin-treated mice.** (**a**) Average expression levels (rapamycin versus control) from chromosomes. RNA-seq was performed on RNA from pachytene spermatocytes isolated from adult control (n=7) and rapamycin-treated (n=15) mice using the STA-PUT method. (**b**) Quantitative RT-PCR analysis of X and Y-linked genes in pooled samples of pachytene spermatocytes used in (a). Levels of *Arbp* mRNA were used as loading control. *P<0.05, ** P<0.01. Student’s t test. (**c-d**) Mean expression levels of autosomal (A), X-linked (X) and Y-linked (Y) genes in control pachytene spermatocytes (**c**) and round spermatids (**d**). (**e**) Average expression levels (rapamycin versus control) for each chromosome in round spermatids isolated from adult mice (control, n=7; rapamycin, n=15). *P<0.05, ** P<0.01.

To evaluate whether the observed defects in sex body composition in rapamycin-treated germ cells affected the gene expression from the sex chromosomes, which are normally transcriptionally silenced during the pachytene stage, we performed whole transcriptome sequencing of mRNA (RNA-seq) from testis tissue and from purified pachytene spermatocytes and round spermatids to account for differential proportions of these germ cell populations in control and rapamycin-exposed testes. STA-PUT velocity sedimentation yielded highly enriched populations of pachytene spermatocytes (91.7% γH2AX-positive; Supplementary Fig. S1a) and round spermatids (90% positive for the acrosome marker peanut agglutinin [PNA]; Supplementary Fig. S1b ).The average level of gene expression from individual chromosomes did not differ substantially between control and rapamycin-treated pachytene spermatocytes, but average expression levels of X- and Y-linked genes were clearly upregulated in the latter (Fig. 4a). We further confirmed RNA-seq results by quantitative RT-PCR for a subset of essential sex chromosome-linked genes subject to meiotic silencing, including the Y chromosome-linked *Zfy1* and *Zfy2* genes. Failed repression of these two genes during meiosis is associated with spermatogenic arrest at the pachytene stage [56]. Consistent with RNA-seq results, *Zfy1* and *Zfy2* transcript levels were significantly increased (1.8 fold and 1.7fold, respectively) in pachytene spermatocytes from rapamycin-treated testes versus controls (Fig. 4b). We also detected significant upregulation of the Y-linked *Rbmy* and *Ube1y* genes, which are normally silenced in pachynema [57,58], and of the X-linked genes Usp26 (1.7 fold), Tktl1 (2.0 fold), Fthl17 (1.9 fold) and Tex11 (1.5 fold) (Fig. 4b). These results suggest that in rapamycin-treated pachytene spermatocytes, many X-and Y-linked genes escaped meiotic silencing.

Although meiotic silencing of sex chromosomes is largely limited to the pachytene stage of male meiosis [49], transcriptional repression of the X chromosome is retained post-meiotically except for a small set of X-linked multi-copy genes [59,60]. Consistent with previous reports, we observed that X-linked genes remained repressed in isolated control round spermatids, whereas transcription of Y-linked genes was detectable (Fig. 4c, d) [61]. However, in rapamycin-treated round spermatids that escaped from pachytene arrest, transcript levels of X-linked genes were clearly upregulated (Fig. 4e). Abnormalities in postmeiotic X chromatin repression have been previously linked with sterility due to the formation of morphologically abnormal haploid spermatids [62]. Our findings therefore suggest that the spermiogenic defects in rapamycin-treated testes are caused by impaired repression of X chromosome activity.

### Chronic rapamycin exposure alters transcription of genes involved in mitochondrial respiration and piRNA metabolism

**Figure 5:**
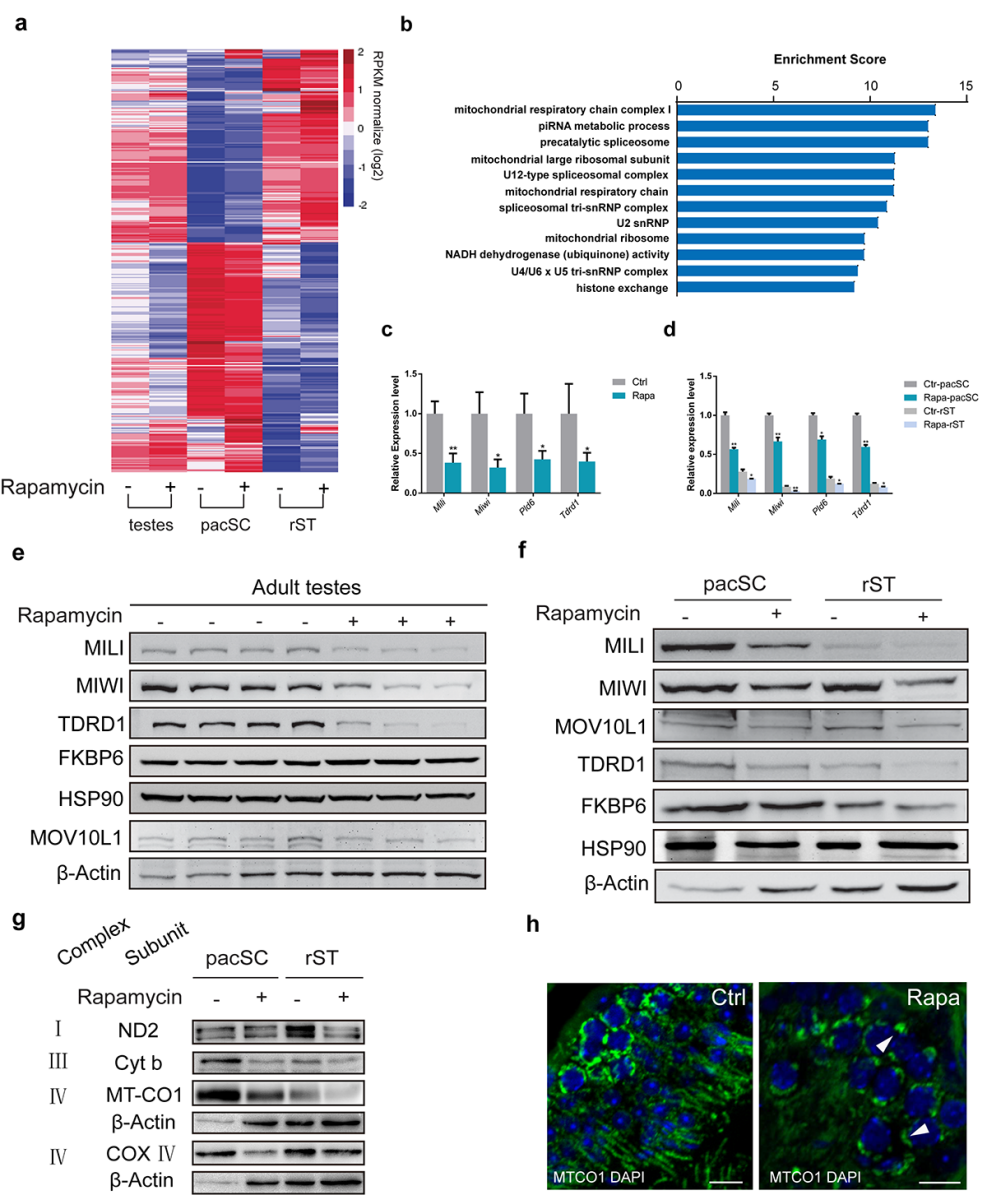
**Chronic rapamycin treatment affects the expression of mitochondrial and piRNA pathway genes.** Samples were testis tissue (3 control, 3 rapamycin-treated males) and pachytene spermatocytes (pacSC) and round spermatids (rST) isolated from pooled samples (control, n=7 males; rapamycin, n=15). (**a**) Heatmap of differentially expressed genes. (**b**) Gene ontology analysis of genes that were downregulated in pachytene spermatocytes from rapamycin-treated mice versus control. (**c**) Quantitative RT-PCR analysis of piRNA pathway component transcripts in testis tissue. Error bars represent SD (*P < 0.05, Student’s t test). (**d**) Relative mRNA level of piRNA pathway genes in pachytene spermatocytes and round spermatid populations. Error bars represent SD (*P < 0.05, Student’s t test). (**e, f**) Western blot analysis of piRNA pathway associated proteins in testis (**e**), and isolated spermatocytes and round spermatids (**f**) from control (-) and rapamycin-treated (+) males. (**g**) Western blot analysis of OXPHOS subunits. β-Actin served as loading control. (**h**) Immunostaining of testis sections for MT-CO1 (green). Arrowheads mark accumulation of MT-CO1 at the periphery of nuclei in rapamycin-treated testis. Scale bar, 20μm.

Gene ontology analysis of genes downregulated in rapamycin-treated spermatocytes using gene ontology classification resulted in a significant enrichment of pathways relating to the mitochondrial respiratory chain and piRNA metabolic processes (Fig. 5a, b). piRNA pathway-related genes with significant downregulation in rapamycin-treated pachytene spermatocytes and round spermatids included the essential piRNA pathway genes Miwi, Tdrd1, Mili, as well as MitoPLD (PLD6) and Gasz, two mitochondrial genes involved in nuage formation and piRNA biogenesis [63–65] (Fig. 5c, d). Western blot analysis confirmed substantially lower levels of MIWI, MILI, and TDRD1 in whole testes and purified cell populations from rapamycin-treated mice compared with controls, whereas the levels of other proteins such as FKBP6 and HSP90 were similar (Fig. 5 e, f). However, we cannot rule out the possibility that the reduced protein levels of piRNA factors in rapamycin-treated testes could be a consequence of altered cell types resulting from arrested germ cell development. Thus, in addition to the defects in silencing sex chromosomes-linked genes in rapamycin-treated pachytene spermatocytes, RNA-seq data analysis also revealed a subset of genes encoding for piRNA pathway components was decreased.

Other gene groups subject to downregulation in rapamycin-treated testes included a large group of genes with function in the mitochondrial respiratory chain, and genes associated with mitochondrial ribosome (Fig. 5b and Supplementary Fig. S2a). This is consistent with previous studies showing that rapamycin impairs mitochondrial biogenesis and oxidative function in cultured differentiated myotubes and muscle [66–68]. Western blot analysis of mitochondrial-encoded OXPHOS subunits confirmed reduced levels of individual subunits of respiratory complexes I, III and IV in germ cells from rapamycin treated mice compared with controls (Fig. 5g). The most prominent reduction was seen for the MT-CO1 subunit of complex IV, which exhibited a reduction in protein amounts by 40% (Supplementary Fig. S2b). Immunostaining of pachytene spermatocytes for cytochrome C (Supplementary Fig. S2c) and MT-CO1 (Fig. 5h) revealed not only lower levels of these mitochondrial proteins in rapamycin-treated spermatocytes but also abnormal perinuclear clustering.

### Chronic rapamycin exposure reduces the piRNA content of male germ cells

**Figure 6:**
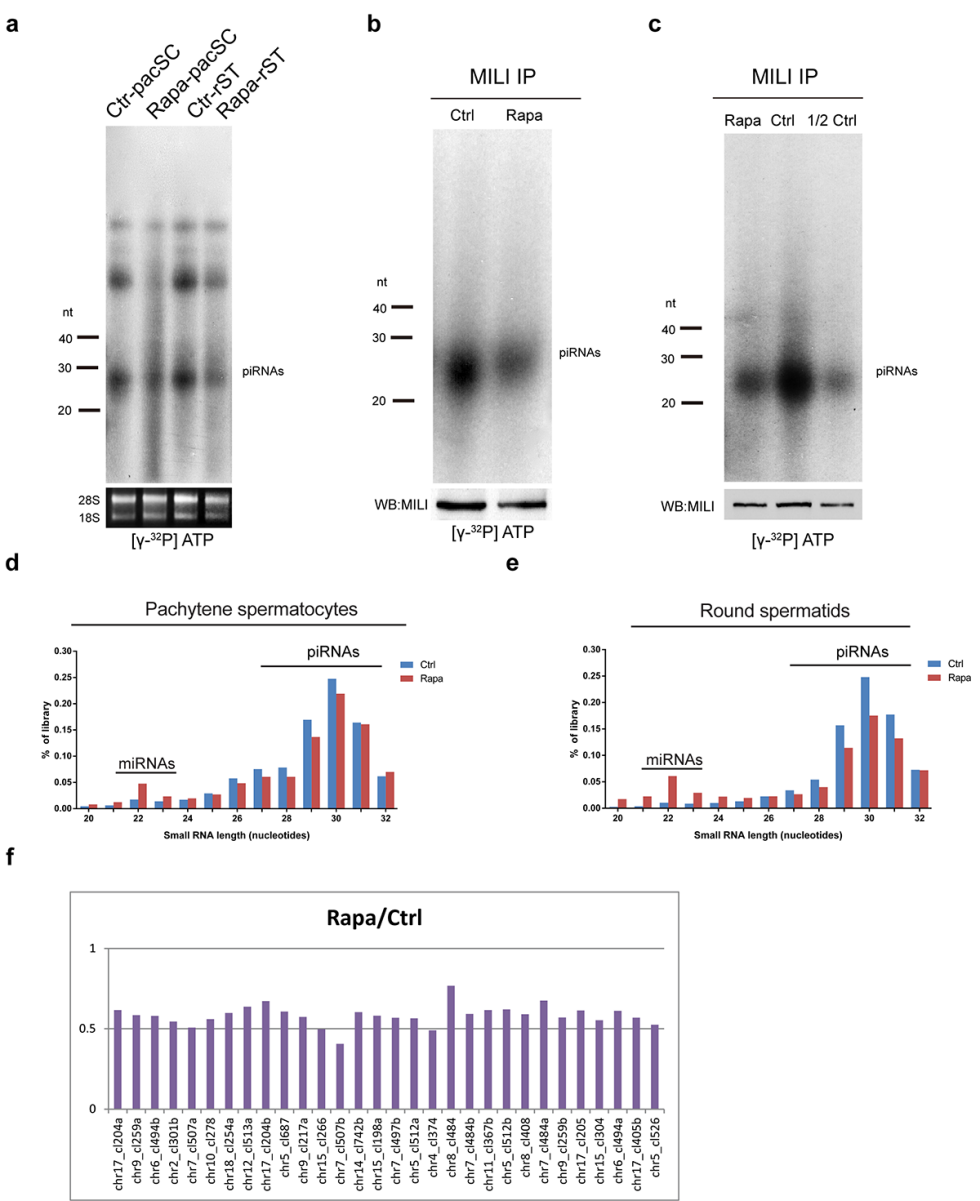
**Chronic rapamycin treatment decreases piRNAs in mouse testes.** (**a**) Reduction of piRNAs in isolated pachytene spermatocytes and round spermatids from adult rapamycin-treated mice. Levels of 18S and 28S ribosomal RNAs were used as a loading control. (**b**) Immunoprecipitation (IP) of MILI-associated piRNAs from control and rapamycin-treated testis followed by ^32^P-end-labeling. MILI protein levels were determined by western blotting. (**c**) MILI-IP complexes from control testes lysate in (**b**) were diluted (1:2). (**d, e**) Length distribution in total small RNA libraries generated from isolated pachytene spermatocytes (d) and round spermatids (e). (**f**) Levels of cluster-derived small RNA reads (24-32nt) in rapamycin-treated relative to control round spermatids. Reads were mapped to pachytene piRNA clusters and the top 30 piRNA clusters producing the largest amounts of piRNAs are shown.

To evaluate the impact of rapamycin on piRNA populations, we radiolabeled total small RNAs isolated from pachytene spermatocytes and round spermatids from control and rapamycin-treated testes. As shown in Fig. 6a, both cell types contained substantially lower piRNA levels compared with controls. Immunoprecipitation of the piRNA-associated protein MILI from rapamycin treated testes revealed that MILI-bound piRNA species were drastically reduced (Fig. 6b). However, when accounting for the difference in MILI protein levels by diluting control IP complexes by half, the levels of MILI-associated piRNAs were comparable between control and rapamycin-treated testes (Fig. 6c), suggesting that the reduction of MILI-bound piRNAs in the rapamycin-treated cells was directly related to reduced availability of MILI protein. We next performed deep sequencing of small RNA populations of 15-32nt length from sorted pachytene spermatocytes and round spermatids and found a substantial reduction in the proportion of piRNA populations in rapamycin-treated samples versus controls (Fig. 6d, e). Upon initiation of meiosis, piRNA populations in the testis change from primary and secondary species, which are enriched for transposon content, to primary piRNAs, which are transposon-poor and derived from distinct pachytene piRNA clusters [25,27,39]. The levels of piRNA species with transposon content were similar between control and rapamycin-treated adult testes (Supplementary Fig. S3a), but the latter contained lower levels of piRNAs from the top 30 piRNA clusters which produce the majority of pachytene piRNAs (Fig. 6f). Consistent with normal levels of transposon-derived piRNAs, there was no evidence for upregulation of Line1 retrotransposons or L1-encoded ORF1P (Supplementary Fig. S3b-d). Upregulation of L1-encoded ORF1P has been previously observed in mice with whole-body ablation of *Mov10l1,* a gene that is implicated in the primary processing of piRNAs (Supplementary Fig. S3d) [69]. However, activation of Line1 was not detected in the testis from mice with conditional deletion of *Mov10l1* during meiosis [70].

### Rapamycin-induced spermatogenic arrest is reversible

**Figure 7:**
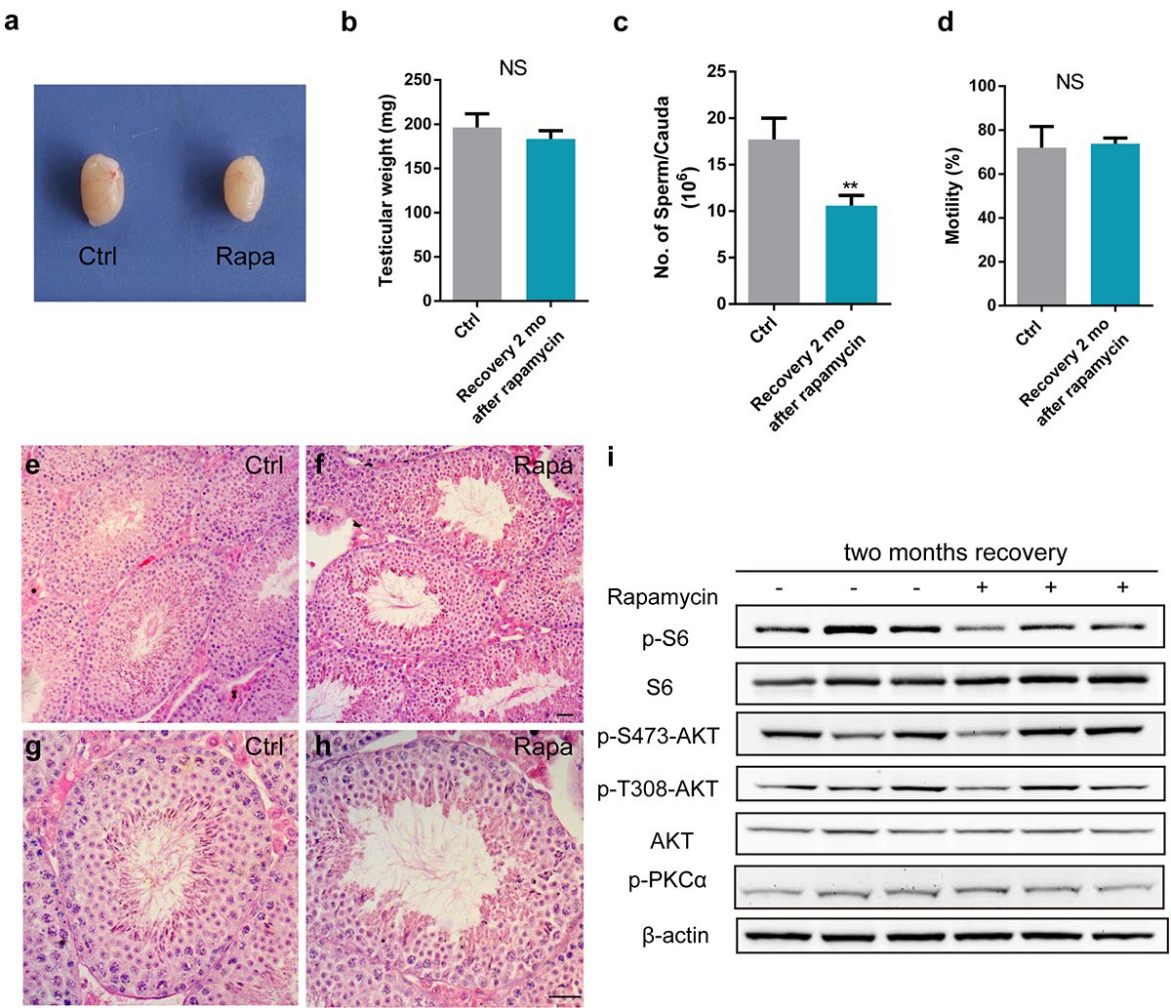
**Rapamycin causes a reversible effect on spermatogenesis.** All panels show samples from adult males injected with vehicle control (ctrl-rec, n=3) or rapamycin for 3 weeks (rapa-rec, n=3), followed by a recovery period of 2-months. (**a**) Gross testis morphology. **(b-d**) Testis mass (**b**), sperm count (**c**), and sperm motility (**d**). Data represent the mean ±SD (NS, not significant, **P<0.01, Student’s t test). (**e-h**) Testis histology reveals full spectrum of spermatogenesis at 2 months after rapamycin treatment cessation. (**i**) Western blot analysis of phosphorylation of mTORC1 and mTORC2 substrates.

Rapamycin and its analogs have been approved to treat cancer and for use as immunosuppressants in organ transplantation. Importantly, rapamycin is the only molecule that extends the life span of genetically heterogeneous mice [13]. Therefore, we examined whether chronic rapamycin treatment-induced spermatogenetic arrest and male infertility were reversible, by transiently exposing mice to rapamycin treatment for 3 weeks, followed by a recovery period of 2 months. Testes from mice exposed to transient rapamycin treatment did not differ in size and weight from controls (Fig. 7a, b). Compared with sperm parameters of rapamycin treated-mice without recovery, sperm counts were increased, and sperm motility was normal (Fig. 7c, d). A full spectrum of spermatogenetic cells was present in testis tubules at 2 months post rapamycin discontinuation (Fig. 7e-h). Western blot analysis of testis tissue 2 months after rapamycin discontinuation showed similar mTOR signaling activity as in controls, with comparable levels of the mTORC1 substrate phospho-S6 and phosphorylated mTORC2 substrates, including AKT S473 and protein kinase PKCα S657 (Fig. 7i). These results suggest that the inhibitory effect of rapamycin on spermatogenesis and mTOR complexes is not permanent.

## DISCUSSION

Here, to study the potential function of mTOR signaling in male germ cell development, we used chronic rapamycin treatment at approximately the same dose that was used to extend lifespan, and found that rapamycin-mediated prolonged inhibition of mTOR signaling causes male infertility, specifically, meiotic defects resulting from disruption in the meiotic silencing of sex chromosomes. Further analysis revealed that the recruitment of the essential silencing factor ATR to the sex chromatin was attenuated at the pachytene stage. ATR is a key mediator of meiotic silencing, and it is a member of the phosphatidylinositol-3 kinase-like kinase (PIKK) family which also includes mTOR [49]. Importantly, ATR catalyzes H2AX phosphorylation, regulates localization of a set meiotic silencing components at unsynapsed axes, and is required to induce repressive epigenetic modifications [53]. We recently showed that the accumulation of ATR on sex chromatin was also diminished in pachytene spermatocytes from testis-specific *Raptor* (mTORC1 subunit) knockout mice, which arrested at pachytene stage with defects in meiotic sex chromosome inactivation [71]. However, meiotic progression and recruitment of silencing factors to sex chromosomes was normal in testes with conditional knockout of mTORC2 component *Rictor* [72] (Supplementary Fig. S4 ). These results suggest that rapamycin-mediated defects in meiosis and meiotic silencing of sex chromosomes is mTORC1-dependent. Previous studies have reported that rapamycin inhibits the proliferation of cultured mouse spermatogonial stem cells (SSCs) [16], but it strengthens the self-renewal potential of undifferentiated spermatogonia in mice with conditional ablation of *Plzf,* a transcription factor essential for the maintenance of undifferentiated spermatogonia [73,74]. Rapamycin has also been found to reduce testis size by blocking the differentiation of spermatogonia in neonatal mice at P4 and P8 [17]. This mechanism is unlikely to be important in our adult mouse model because long-term rapamycin treatment did not affect the level of leptotene spermatocytes but specifically late pachytene and diplotene spermatocytes. Furthermore, the initial recruitment of DNA damage pathway proteins at the leptotene/zygotene stage and the formation of early recombination intermediates occurred normally in rapamycin-treated testes. However, this mechanism may play a role in the shorter-term effects of rapamycin on germ cell development in neonatal testes, when spermatogonial cells are enriched.

In addition to inappropriate expression of sex-linked genes and the associated meiotic defects, we were surprised to find that a disproportionate number of genes for piRNA pathway and mitochondrial respiratory chain were deregulated, indicating that chronic exposure to rapamycin exerts an influence on piRNA populations. Previous studies have shown that mTOR signaling regulates small RNA homeostasis in somatic cells. Observations in murine embryonic fibroblasts and cancer cells showing that modulation of mTOR signaling affected miRNA profiles by altering Drosha activity have previously implied a connection between mTOR and miRNAs [20]. In *C. elegans*, the piRNA pathway regulates germline-specific alternative splicing of TOR (let-363), a homolog of mammalian mTOR [24]. Here we found that in rapamycin-treated testes, miRNA populations were only minimally perturbed and expression of miRNA pathway components was not detectably altered. In contrast, we found reduced expression of genes involved in the piRNA pathway and mitochondrial biogenesis, consistent with previous observations that mTOR signaling positively regulates mitochondrial oxidative function and mitochondrial biogenesis in myotubes [67,75]. Abnormal distribution of mitochondria has been linked to concomitant aberrant distribution of piRNA pathway components, such as in *Mov10l1* and *Mitopld* mutant mouse model [64,65,69]. Thus, impaired mitochondrial biogenesis and abnormal distribution of mitochondria may disturb piRNA populations in rapamycin-treated testes in addition to reduced transcript and protein levels of piRNA factors including MILI, MIWI and TDRD1. Changes in piRNA abundance may in part also be an indirect consequence of arrested germ cell development resulting from rapamycin treatment. Although rapamycin treatment reduced the levels of piRNAs and piRNA pathway proteins in the testis, there was no effect on LINE1 RNA levels. In adult mouse testes, pachytene piRNAs compose >95% piRNAs and are predominantly derived from non-repetitive pachytene piRNA clusters [32,35]. Previous studies have also shown that the slicer activity of MIWI directly cleaves transposon messenger RNAs, which is piRNA amplification-independent [76]. Moreover, the silencing of LINE1 requires multiple epigenetic mechanisms during adult spermatogenesis [77]. It is therefore possible that the levels of active MILI and MIWI in rapamycin-treated testes was sufficient to silence LINE1 transposons.

Taken together, our investigations have demonstrated important roles of mTOR in meiotic progression and male germ cell development through regulation of meiotic silencing sex chromosomes-linked genes and piRNA biogenesis, although the direct downstream target for mTOR signaling in these events remains unclear. Chronic rapamycin treatment inhibits the recruitment of ATR, an essential silencing factors, to the XY chromatin at pachytene stage. The repression of many sex-linked genes fails, concomitant with altered establishment of repressive epigenetic marker on the sex chromosomes. Further, chronic rapamycin exposure disturbs the homeostasis of noncoding RNAs by reducing the piRNA population. Finally, we provide evidence that rapamycin reversibly targets male germ cells, because, following a recovery period, testes weight, histological characteristics and sperm numbers of rapamycin-treated males recover to the levels of controls. Our study will help to evaluate the consequences of chronic rapamycin use for the treatment of cancers and age-related diseases.

## MATERIALS AND METHODS

### Animals

All mice were maintained and used for experimentation according to approved Institutional Animal Care and Use Committee protocols of Nanjing Medical University under the accession number 1403021. All mice were kept on 12 h light/ 12 h dark cycle, fed with a standard chow diet and housed in a temperature-controlled, pathogen-free barrier facility. Male C57BL/6 mice were obtained from Vital River Laboratories at approximately 7 weeks of age. For chronic rapamycin treatment, 7- to 8-week-old old were injected intraperitoneally once daily with rapamycin (2mg/kg) or saline vehicle for 3 weeks. Rapamycin was purchased from LC Laboratories.

### Quantitative Real Time RT-PCR

Total RNA was extracted from samples using TRIzol reagent, and concentration and purity of RNA determined by absorbance at 260/280 nm. 1 µg of total RNA was reverse transcribed using a high-capacity cDNA reverse transcription kit (Applied Biosystems) according to the manufacturer’s instructions, and the cDNA was subject to real time PCR using SYBR Q-PCR master mix (Applied Biosystems). A typical reaction contained 250 nmol/l of forward and reverse primer, 1 µl cDNA in a final reaction volume of 20 µl. The reaction was initiated by preheating at 50°C for 2 min, followed by 95°C for 10 min. Subsequently, 40 amplification cycles were carried out with 15 s denaturation at 95°C and 30s annealing and extension at 60°C. Gene expression was normalized to *Arbp*. PCR primers were as follows: *Usp26*, F: AATGTAACGAAGGGAGAAGTG, R: AGGCTTTGCCTTCTTATCGAG; *Tktl1*, F: TCAAAGGGACTACCATTTGTT, R: AACAGGGGGCGAAGTCATACA; *Rbmy*, F: ACCATCCTTTTCAAGAACCAGA, R: TAACTGCAAAGTGTCTCCCAGA; *Ube1y*, F: ATTGACTTTGAGAAGGATGAC, R: CAGACACACAAGGCCAACTAT; *Zfy1*, F: CAGATCAGAGCACTAGCATTCG, R: CTGGCAGTGACATTCTGGTCT; *Zfy2*: F: ATCCTTTGACAGCCGACATTT; R: CCTCACAGTTGATTCTGGCATC; *Fthl17*, F: TCTCGAATGCAGCAGAACTATG, R: GGTCAAAGTAGACTGCCATCG; *Tex11*, F: GACTGTGGGGTATTGCTTCTG, R: CAACTGGCTCCTGTTTTCTGT; *Arbp*, F: GCAGATCGGGTACCCAACTGTTG, R: CAGCAGCCGCAAATGCAGATG.

### Immunoblotting

Cells were rinsed with PBS and lysed in cold RIPA buffer supplemented with phosphatase inhibitor and protease inhibitor cocktail tablets (Roche). Cell lysates were incubated on ice for 10 minutes, sonicated on ice for 30 seconds, and centrifuged at 12,800 rpm for 15 minutes at 4°C. Protein concentration was determined by Bicinchoninic Acid (BCA) Assay (Pierce Biotechnology). 20ug protein were separated by sodium dodecylsulphate-polyacrylamide gel electrophoresis (SDS-PAGE) on 8-16% gradient or 10% resolving gels. Quantification was performed by densitometry using ImageJ software, and loading was verified by blotting for Actin as indicated. Supplementary Table S1 lists all primary antibodies used.

### Immunoprecipitation

Tissue samples were homogenized in cold 0.3% CHAPS lysis buffer (40mM Hepes [pH 7.5], 120mM NaCl, 1mM EDTA, 0.3% CHAPS, 10mM Pyrophosphate, 10mM β-glycerophosphate, 50mM NaF, 0.5mM Orthovanadate, and protease inhibitors). Cell and tissue lysates were incubated at 4°C for 15min or 1hr, respectively, followed by centrifugation at 16,000 rpm at 4°C for 15 min and 30min, respectively, to discard insoluble material. Protein A agarose beads were added to the supernatant and samples incubated on a nutator for 1hr, followed by removal of the beads by centrifugation and addition of mTOR antibody to the pre-cleared lysates overnight at 4°C. Protein A agarose beads were added into the supernatant and incubated at 4°C for another 3hr. Immunoprecipitated complexes with protein A agarose beads were washed by 0.3% CHAPS lysis buffer three times, boiled in SDS-sample buffer, separated on 10% SDS-PAGE, and analyzed by immunoblotting. This method is modified from that described by Sarbassov [78].

### Immunoprecipitation of small RNAs

Mouse testes were homogenized and tissue lysates were immunoprecipitated with MILI antibody. MILI associated small RNAs were extracted and purified. 5’-end labelled small RNAs were resolved by 15% urea-PAGE as described previously [79].

### Histology and TUNEL staining

For histology, testes were fixed in Bouin’s solution (Sigma) overnight, dehydrated in ethanol, embedded in paraffin, sectioned, and stained with hematoxylin and eosin. TUNEL staining was performed on frozen sections using the ApopTag Fluorescein in Situ Apoptosis Detection kit (Milipore,2654350) kit according to the manufacturer's instructions [80].

### Immunofluorescence, chromosome spread, and electron microscopy (EM) analyses

Immunofluorescence was performed on frozen sections of testes. Briefly, testes were fixed in 4% paraformaldehyde, dehydrated in sucrose and embedded in OCT as previously described [69]. Primary antibodies used for immunofluorescence are listed Supplementary Table S1. EM was performed at the Biomedical Imaging Core facility a Nanjing Medical University. Briefly, testes were fixed, dehydrated, embedded and polymerized by automated microwave tissue processor (Leica EMAMW). After polymerization, sections were prepared using a RMC ultramicrotome and photographed with a Transmission Electron Microscope (JEM-1010).

### mRNA deep sequencing, small RNA sequencing and bioinformatic analyses

Pachytene spermatocytes and round spermatids were isolated from adult control and rapamycin-treated mice (n=7-15). Testes from *Raptor^cko^* and *Rictor^cko^* mice were collected at postnatal day 18. Total RNA was extracted from samples using TRIzol reagent. RNA samples were from three different mice (n=3 per genotype). Per sample, three RNAs with an A260:A280 ratio of 1.8 to 2.0 were pooled and applied for sequencing. Strand-specific libraries were prepared using the TruSeq Stranded Total RNA Sample Preparation kit (Illumina, USA) according to the manufacturer's instructions. Sequencing was performed on an Illumina Hisequation 2500 system. The q value cut-off for significantly different gene expression was set to P<0.05. DAVID Functional Annotation tool was used for ontology analysis. For small RNA sequencing, small RNAs ranging from 15 to 32nt in size were purified by gel extraction, and small RNA libraries were prepared and deep-sequenced using the Illumina Solexa technology. Only sequences reads that mapped to the genome (mm9) were considered for analysis. Cluster analysis of intergenic piRNA hotspots (piRNA clusters) was performed as described previously [35]. For reads mapping to transposon consensus sequences from RepeatMasker, three differences or mismatches were allowed. Library construction and sequencing were performed at Shanghai Biotechnology Corporation. We loaded mRNA deep sequencing and small RNA sequencing data to NCBI database, and the sequencing reads are obtained from the National Center for Biotechnology Information's SRA database under the accession number PRJNA421857.

### Statistical analysis

All data are reported as mean ± S.D. unless otherwise noted in the figure legends. Significance was tested by using the 2-tailed unpaired Student's t test (*p < 0.05; **p < 0.01; ***p < 0.001) using Prism 7.0 (GraphPad Software, La Jolla, CA, USA). For the quantification of meiotic silencing factors, χ^2^ test (Prism 7.0) was used to reveal the statistical differences between control and rapamycin group.

## SUPPLEMENTARY MATERIAL

Supplemental File
