# Supplementary material for: Rapamycin-mediated mTOR inhibition impairs silencing of sex chromosomes and the pachytene piRNA pathway in the mouse testis
Source: Aging (Albany NY). 2019 Jan 13;11(1):185–208. doi: 10.18632/aging.101740 (PMC6339782; doi:10.18632/aging.101740)
Supplement: Supplemental File [file aging-11-101740-s001.pdf]

SUPPLEMENTARY MATERIAL

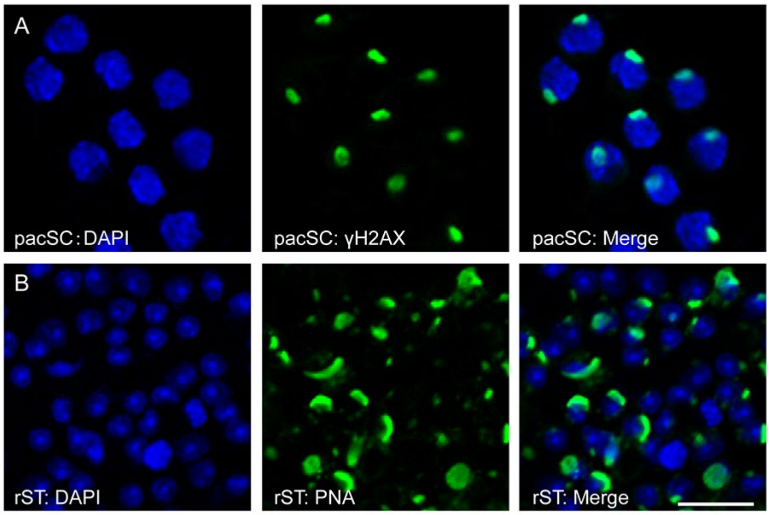

Supplementary Figure S1. Purities of isolated mouse spermatogenic cells. Pachytene spermatocytes (pacSC) and round spermatids (rST) were isolated from adult testes by STA-PUT method. Pachytene spermatocytes were immunostained with their specific marker protein γH2AX, which specifically stains XY body (**a**), and isolated round spermatids were immunostained with PNA for acrosome (**b**).

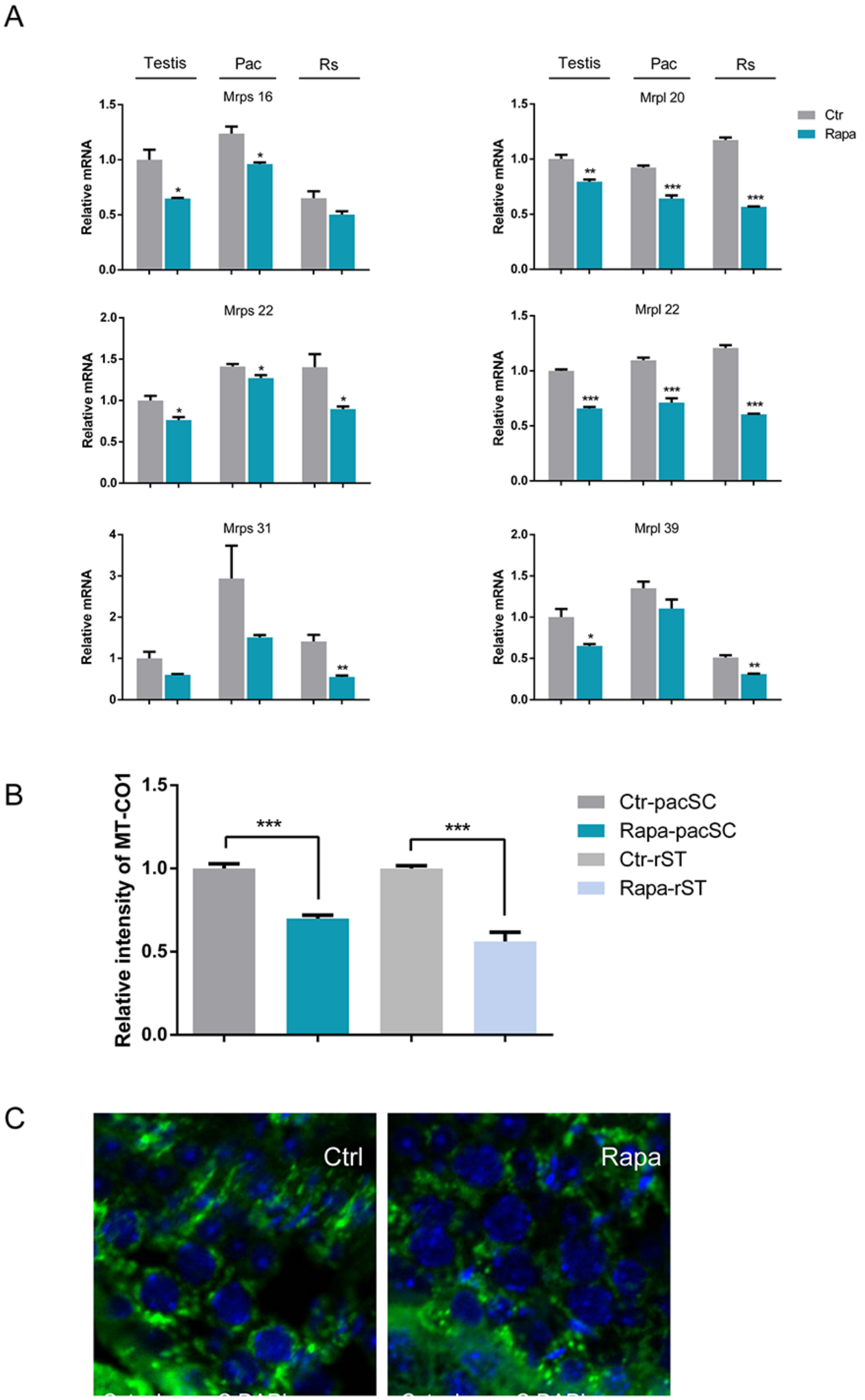

Supplementary Figure S2. Mitochondrial proteins and genes encoding for mitochondrial ribosome were decreased. (**a**) Relative expression levels of genes for mitochondrial ribosome in whole testes and isolated cell populations by quantitative RT-PCR (testes, n=3-4, isolated spermatocytes, n=7-15, *P<0.05, ** P<0.01, *** P<0.001). (**b**) Quantification of OXPHOS subunit MT-CO1 in isolated cell populations (Error bars represent SD, *** P<0.001).

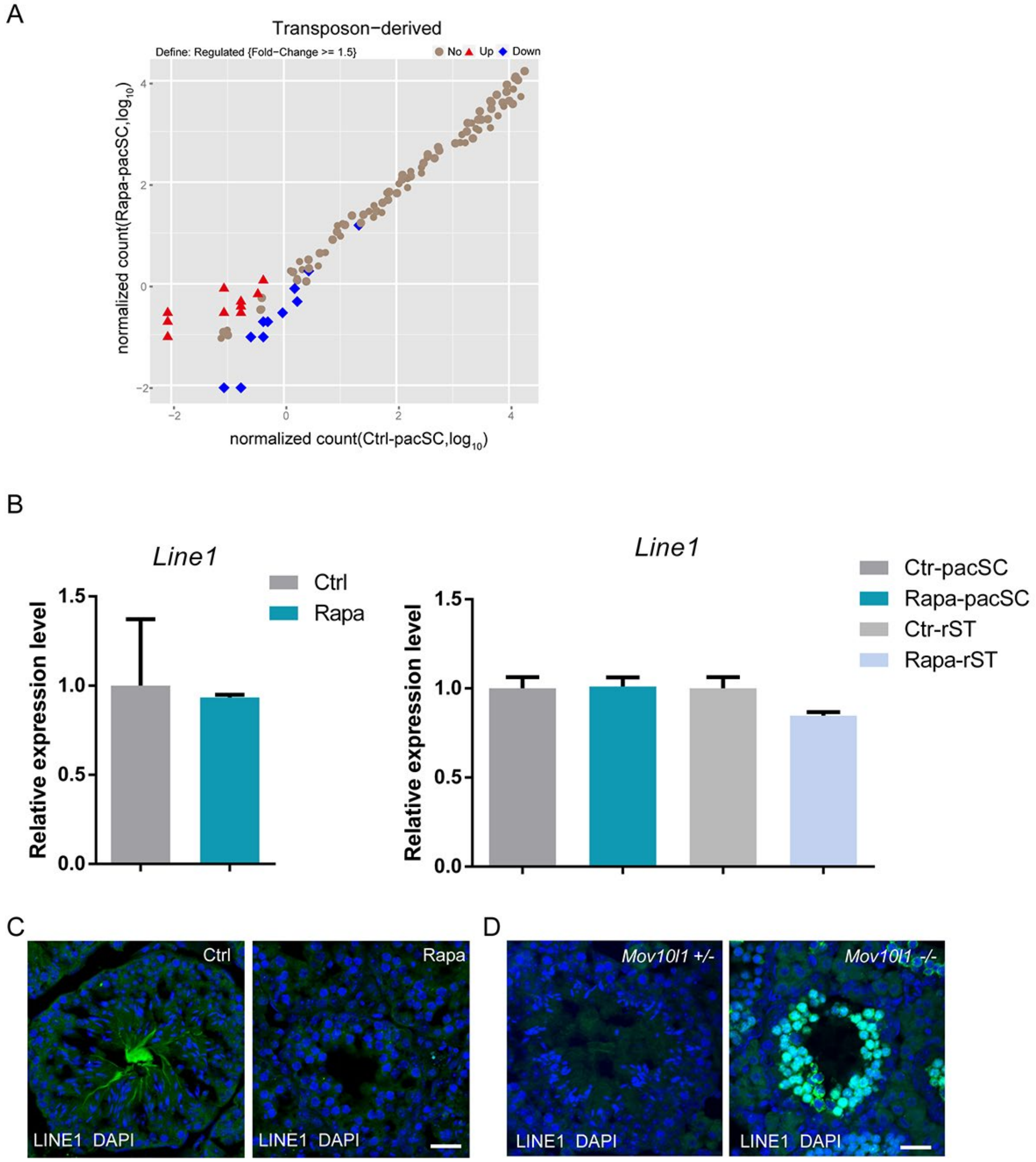

Supplementary Figure S3. Transposons remained similarly expressed in adult testes and isolated cell populations. (**a**)Small RNA sequence reads (24-32nt) that mapped to the mouse transposons sequence in pachytene spermatocytes from adult rapamycin-treated testes (vertical axis) relative to those from control testes (horizontal axis). (**b**) Quantitative RT-PCR analysis of Line1 in testes and isolated cell populations. (**c**) Immunostaining of LINE1 ORF1p (green) on testes sections from adult control and rapamycin-treated mice. (**d**) Immunostaining of LINE1 ORF1p (green) in wild-type (*Mov10l1*$^{+/-}$) and whole-body *Mov10l1* knockout (*Mov10l*$^{-/-}$) testes at postnatal day 60. Scale bar, 20µm.

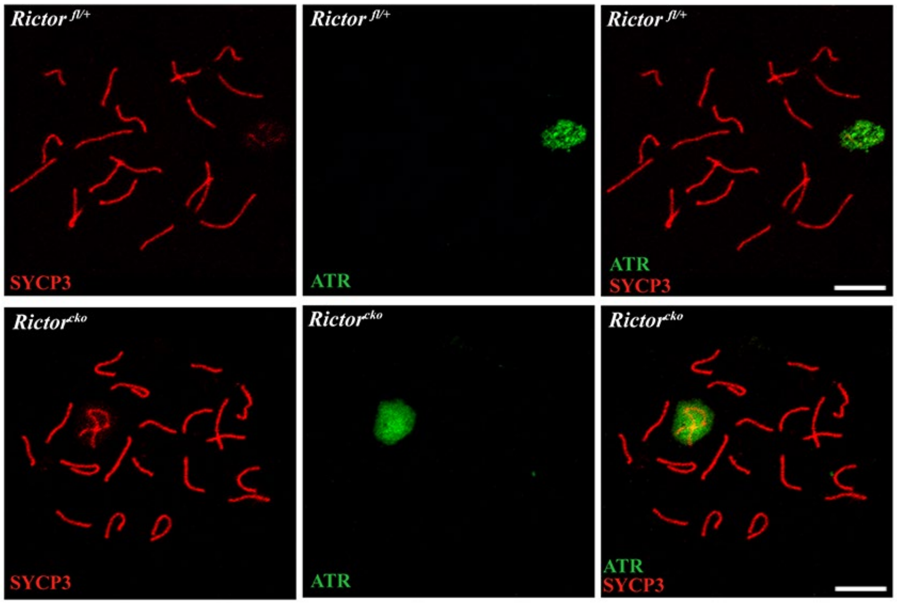

Supplementary Figure S4. Normal distribution of the sex body silencing factor ATR in pachytene spermatocytes from testis-specific *Rictor* knockout (*Rictor*^{cko}) mice. Spread nuclei of spermatocytes from wild-type and *Rictor*^{cko} testes were prepared followed by immunolabeling of ATR (Green) and the synaptonemal complex protein SYCP3 (Red). Wild-type and *Rictor*^{cko} mice were at 35-day-old (n=50 for each genotype). Scale bar, 10 µm.

Supplementary Table S1.

Antibody Name	Manufacturer	Application
Anti-MILI	Abcam,ab36764	WB
Anti-MIWI	Abcam,ab12337	WB
Anti-TDRD1	gift from Dr. Shinichiro Chuma	WB
Anti-PLD6	gift from Dr. Watanable Toshiaki	WB
Anti-FKBP6	Abcam,ab108419	WB
Anti-ND2	Abcam,ab102753	WB
Anti-Cytochrome b	Abcam,ab182330	WB
Anti-ATP6	Abcam,ab192423	WB
Anti-MT-CO1	Abcam,ab14705	WB/IF
Anti-LINE1 ORF1p	gift from Dr. Ramesh Pillai	WB/IF
Anti-phospho-Akt S473	Cell signaling,4058S	WB
Anti-phospho-Akt T308	Cell signaling,9275S	WB
Anti-AKT	Cell signaling,9272S	WB
Anti-phospho-S6 ribosomal protein	Cell signaling,2215S	WB
Anti-S6 ribosomal protein	Cell signaling,2217S	WB
Anti-p-4EBP1 S65	Cell signaling,9451S	WB
Anti-phospho-NDRG1 T346	Cell signaling,3217S	WB
Anti-mTOR	Cell signaling,2972S	WB
Anti-Raptor	Cell signaling,2280S	WB
Anti-Rictor	Cell signaling,2140S	WB
Anti-COXIV	Cell signaling,4850S	WB
Anti-phospho-PKCα	Santa Cruz,sc12356-R	WB
Anti-β-Actin	Sigma, A5316	WB
Anti-MILI	gift from Dr. Ramesh Pillai	IF
Anti-MIWI	gift from Dr. Ramesh Pillai	IF
Anti-Cytochrome C	Abcam, ab110325	IF
Anti-γH2AX	Millipore, 16-202A	IF/Spread
Anti-SYCP1	Abcam, ab15090	Spread
Anti-SYCP3	Abcam, ab97672	Spread
Anti-ATR	Santa Cruz, sc1887	Spread
Anti-Rad51	Santa Cruz, sc8349	Spread
Anti-MLH1	BD,550838	Spread
Anti-H3K9me3	Abcam, ab8898	Spread
